# Biodegradation of Hard Keratins by Two *Bacillus* Strains

**DOI:** 10.5812/jjm.8896

**Published:** 2014-02-01

**Authors:** Wojciech Laba, Anna Rodziewicz

**Affiliations:** 1 Department of Biotechnology and Food Microbiology, Wroclaw University of Environmental and Life Sciences, Wroclaw, Poland

**Keywords:** *Bacillus polymyxa*, *B. cereus*, Keratinases, Proteases, Keratin Biodegradation

## Abstract

**Background:**

Extensive quantities of keratinic by-products are disposed annually by animal-processing industry, causing a mounting ecological problem due to extreme resilience of these materials to enzymatic breakdown. There is a growing trend to apply cheap and environment-friendly methods to recycle keratinic wastes. Soil bacteria of profound keratinolytic potential, especially spore-forming rods from the genus *Bacillus*, play a significant role in keratinase-mediated biodegradation of keratins, therefore could be effective in hastening their biodegradation. Keratin hydrolysis in microbial cultures is one of the most promising techniques not only to utilize this protein but also to obtain valuable by products.

**Objectives:**

The study was undertaken to investigate the biodegradation process of various keratinic materials by two *Bacillus* strains.

**Materials and Methods:**

Two keratinolytic strains, *Bacillus cereus* and *B. polymyxa*, were subject to cultures in the presence of several keratinic appendages, like chicken feathers, barbs and rachea of ostrich feathers, pig bristle, lamb wool, human hair and stratum corneum of epidermis, as main nutrient sources. Bacterial ability to decompose these waste materials was evaluated, at the background of keratinase and protease biosynthesis, in brief four-day cultures. Keratinolytic activity was measured on soluble keratin preparation and proteases were assayed on casein. Additionally, amounts of liberated proteins, amino acids and thiols were evaluated. Residual keratin weight was tested afterwards.

**Results:**

Both tested strains proved to be more adapted for fast biodegradation of feather β-keratins than hair-type α-keratins. *B. cereus* revealed its significant proteolytic potential, especially on whole chicken feathers (230 PU) and stratum corneum (180 PU), but also on separated barbs and rachea, which appeared to be moderate protease inducers. Keratinolytic activity of *B. cereus* was comparable on most substrates and maximum level obtained was 11 KU. *B. polymyxa* was found to be a better producer of keratinases, up to 32 KU on chicken feathers and 14 KU on both fractions of ostrich feathers. Its proteolytic activity was mostly revealed on stratum corneum and human hair. Stratum corneum was extensively degraded by both bacterial strains up to 99% - 87%, chicken feathers 47-56%, ostrich barbs and rachea, 28% and 35% at maximum, respectively. Keratin fibres of structures like human hair, lamb wool and pig bristle remained highly resilient to this short microbiological treatment, however certain extent of keratinase induction was also observed.

**Conclusions:**

The obtained results prove that keratinolytic potential of both tested bacterial strains could be applied mainly in biodegradation of feathers, however, *B. cereus *and *B. polymyxa* differed in terms of keratinase and protease production on each of the substrates. Biodegradation of highly resilient structures like hair or pig bristle requires further analysis of process conditions.

## 1. Background

Keratinic materials in the form of agro-industrial waste increasingly accumulate in the environment. The extremely resilient nature of this protein gradually leads to problematic ecological issues. Growing demand for efficient alternative to traditional recycling techniques guides towards application of keratinolytic microorganisms in the bioconversion process. Among numerous microbial groups many keratin degraders derive from the bacterial genus *Bacillus*. 

Keratin proteins are the major constituents of epidermal structures, which function is connected with mechanical protection of skin surface. Keratin of epidermal stratum corneum is listed among “soft”, cytoskeletal keratins. Its fibres build the largest group of intermediate filaments in cytoskeleton of epithelial cells, which function exceeds simple mechanical support and is associated with cell-to-cell communication, as well as cell cycle and apoptosis mechanisms (1, 2). Cytokeratins form single-phase structures, unlike “hard” keratins, are more susceptible to proteolytic cleavage. Vertebrate skin appendages are, however, based on two-phase organization, where tightly packed and extensively cross-linked polypeptide chains are embedded in amorphous high-cysteine protein matrix. 

These “hard” keratins, despite high sequence homology to cytokeratins, present unique resilience to mechanical stress and cleavage by common proteases. Mammalian fibres share common structural organization, however certain level of variation between species, or even within hair of a single animal is observed. The structure of hair-type of appendages incorporates a complex multi-layer cuticle and an internal cortex. Outer layers of cuticle, responsible for protection against mechanical and chemical factors, include hydrophobic surface membranes and non-keratinic, high-cysteine epicuticle and exocuticle, followed by low-sulfur endocuticle. The cortex, responsible for mechanical properties of fibres is formed of long, polyhedral cortical cells, including α-keratin intermediate filaments submerged in sulfur-rich matrix, mostly organized into macrofibrils (3).

Avian feathers also present an example of filament-matrix organization. Feather beta-keratins are very little extensible proteins containing β-pleated sheet. The result of the aggregation of beta-keratin molecules produces highly durable filaments and bundles arranged into ultrastructural pattern of 3–4 nm filaments submerged in amorphous matrix. Beta-keratin layers mainly generate mechanical and chemical resistance, thus durability of avian scales, claw, beak, and feathers. Despite most keratins in feathers being proteins of 8–12 kDa, also α-keratins (intermediate filament proteins) of higher molecular weight are present at the beginning of the differentiation of barb, barbules, and calamus cells as well as in adult feather. 

Alpha-keratins comprise proteins of 40–70 kDa made by the aggregation of intermediate 8–10 nm keratin filaments. Two α-keratin types, an acidic type and a basic type, form a heteropolymer, which aggregates into the 8–10 nm-thick intermediate filament. Packets of keratin filaments mainly function for epidermal stretching and they associate with lipids as hydrophobic barrier. The cysteine content according to the amino acid sequence is 7% which allows extensive crosslinking within the protein. Keratin has about 40% of hydrophilic and 60% of hydrophobic amino acid residues in the sequence protein molecules in the fibre of mature feather assemble into α-helix, β-sheet or disordered state, at the ratio 41:38:21 (4-6).

There is a growing trend to apply cheap and environment-friendly methods to recycle keratinic wastes, as an alternative to traditional techniques of utilization or bioconversion, often involving high energy requirement or high concentrations of reducing or oxidative chemicals. Keratin hydrolysis can be performed in microbial cultures, as well as by cell-free keratinase extracts, and still remains one of the most promising techniques not only to utilize this protein but also to obtain valuable products in the form of protein hydrolysates or pretreated intermediates. Keratinolytic microorganisms are often potent producers of proteases exhibiting high specificity towards keratin or other fibrous proteins (7, 8). Nevertheless, keratinolysis is additionally supported by reducing factors like sulfite or other sulfur compounds secreted by microorganisms or disulfide reductase enzymes, each playing a significant role in initial cleavage of disulfide bridges in the substrate (9, 10).

## 2. Objectives

In our preceding research we evaluated keratinolytic potential of two feather degrading bacterial strains: *Bacilluscereus* and *B. polymyxa*, which proved to be very effective in decomposition of raw chicken feathers (11). Their keratinolytic abilities were associated with production of predominantly alkaline serine keratinases optimally active at 50° C in the case of *B. polymyxa* and neutral proteases with optimum activity at 45°C in the case of *B. cereus*. The present study was undertaken to investigate in vitro biodegradation of various hard-to-degrade keratin substrates by these bacteria.

## 3. Materials and Methods

### 3.1. Bacterial Strains and Culture Conditions

The studied bacterial strains were *B.polymyxa* B20 and *B. cereus* B5esz, isolated previously from soil and keratinous wastes, respectively (11). Microbial cultures were carried out in 250 cm^3^ Erlenmayer flasks, in 50 cm^3^ of medium, at 30°C and 170 rpm, for four days. The culture medium consisted of (g/dm^3^): MgSO_4_ (1.0), CaCl_2_ (0.1), KH_2_PO_4_ (0.1), FeSO_4_·7H_2_O (0.01), yeast extract (0.5); initial pH 7.1. Nutrient broth culture (nutrient broth 8.0, glucose 10) of 1.2108 cfu/cm^3^ served as inoculum, used in 1 mL per flask. The main carbon and nitrogen source were keratinous skin appendages (10 g/dm^3^): white chicken feathers, barbs and rachea of white ostrich feathers, pig bristle, lamb wool, human hair and stratum corneum of epidermis. The substrates were prepared by washing and degreasing with methanol-chloroform solution (1:1). 

### 3.2. Analytical Methods

Assays were performed in crude culture fluids after removing feather debris through medium density paper filter and centrifugation at 10000 g, 4°C. The concentration of soluble proteins derived from substrates was determined by the method of Lowry et al. (12). The release of -NH2 groups of amino acids was measured using the method described by Snyder and Sobocinski (13). Liberation of thiol compounds was assayed according to the Ellman’s method (13, 14). 

Keratinolytic activity was determined on soluble keratin preparation, at 40°C and pH 7.5. One unit of keratinolytic activity (KA) was defined as the 0.01 increase of TCA-soluble products absorbance at 280 nm, per 1 cm3 of enzyme in 1 minute. Proteolytic activity was determined using the modified method of Anson on casein, at 30°C and pH 7.5. One unit of proteolytic activity (PA) represented an absorbance increase of 0.01 per 1 cm^3^ of enzyme in 1 minute (11). Residual dry matter of keratinous substrates was determined after separation and drying at 105°C.

## 4. Results 

Keratinolytic properties of *B.polymyxa* B20 and *B. cereu*s B5esz against poultry feathers were reported in previous studies (11). Nevertheless, other keratins present in various vertebrate skin appendages are usually less prone to enzymatic digestion and less available to microorganisms. Both tested bacterial strains cultured in mineral media containing different keratins degraded and utilized those substrates to a different extent which correlated with diverse levels of induced keratinases and less specific proteases. 

**Figure 1.**
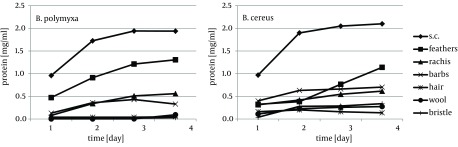
Liberation of Soluble Proteins During Growth of *B. polymyxa* and *B. cereus* in the Presence of Different Keratins

**Figure 2.**
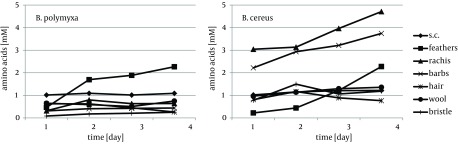
Concentration of -NH2 Groups of Amino Acids in Culture Fluids During Growth of *B. polymyxa *and *B. cereus* in the Presence of Different Keratins

**Figure 3.**
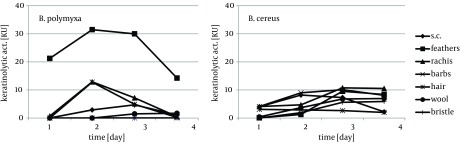
Production of Keratinases During Growth of *B. polymyxa* and *B. cereus *in the Presence of Different Keratins

**Figure 4.**
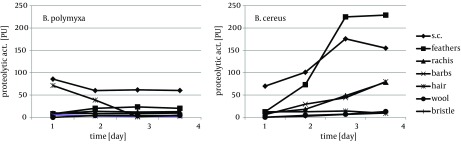
Production of Caseinolytic Proteases During Growth of *B. polymyxa* and *B. cereus* in the Presence of Different Keratins

Cytoskeletal keratin of epithelial origin in the form of native stratum corneum was the most easily decomposed substrate in the presented experiment, where 99.4% - 87.0% loss was recorded after four days of bacterial cultures. As a result, a significant increase of soluble proteins concentration, reaching 1.95 mg/cm^3^ for *B. polymyxa* and 2.10 mg/cm^3^ for *B. cereus*, was observed (Figure 1). It is notable, in case of both strains, that during growth on stratum corneum mainly proteins were extensively accumulated, while concentration of amino acids remained at the level below 1.3 mM. In contrast, in the presence of whole chicken feathers liberation of hydrolysis products, in a form soluble proteins, was lower nearly twice, at the expense of substantial escalation of amino acids (Figure 2). This could be explained either by higher induction of keratinases production in *B. polymyxa* and mainly induction of proteases in *B. cereus*, leading to nearly complete degradation of the substrate. Nevertheless, stratum corneum of epidermis served as a fairly effective inducer of proteases for both strains but moderate inducer of keratinases (Figures 3, 4). 

**Figure 5.**
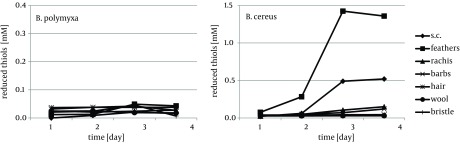
Accumulation of Reduced Thiols in Culture Fluids During Growth of *B. polymyxa* and *B. cereus *in the Presence of Different Keratins

**Figure 6.**
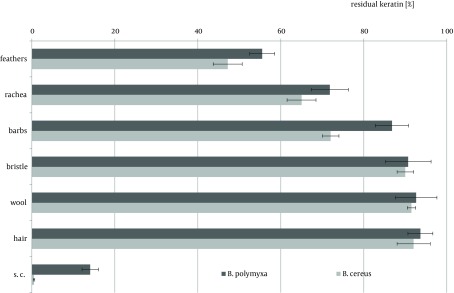
Residual Keratin After 4-Day Cultures of Tested Bacteria

Keratin of chicken feathers is a most abundantly used substrate for production of keratinolytic proteases. Chicken feathers proved to be an appreciable substrate for both tested strains, in terms of keratinase induction, as well as susceptibility to hydrolysis, thus sufficient availability to microorganisms. In the presence of chicken feathers the strain *B. polymyxa* B20 exhibited its highest capability of keratinase production, while *B. cereus *B5esz revealed an exceptional proteolytic potential. As a result, significant substrate deterioration was observed through accumulation of hydrolysis products, including noteworthy amount of reduced thiols in the case *B. cereus* (Figure 5), and a major decrease in residual substrate, reaching from 47.2% to 55.5% after four-day cultures (Figure 6). 

Feather substrate is not completely uniform in structure, however almost entirely composed of β-keratin, due to the presence of rigid rachea and more flexible barbs, which refers especially to flight and contour feathers. The analysis of bacterial cultures on separated feather barbs or rachea, at the example of ostrich feathers, allowed to illustrate dissimilarities within this specific substrate, resulting from different keratin organization. Unexpectedly, hard component of rachis appeared to be a better keratinase inducer, especially for *B. polymyxa*, producing 12.8 KU at maximum. Both fractions, rachis and barbs, were equally moderate substrates for protease production, up to 80 PU for *B. cereus*, however twice less effective in comparison to whole chicken feathers (Figures 3, 4). Furthermore, rachis material was more prone to degradation than barbs, resulting in 7-15% higher substrate loss. 

Hair-type keratinous appendages like pig bristle, lamb wool or human hair comprise a group of extremely resilient structures. The degree of substrate decomposition by both tested strains in four-day cultures did not surpass 10% in neither case and remained within the average content of non-keratinous proteins in these materials (Figure 6). Nevertheless, *B. polymyxa* and *B. cereus* demonstrated diverse capabilities of hydrolytic enzymes biosynthesis in the presence of these materials, despite limited availability of keratin-derived nutrients. 

*B.polymyxa*, unlike *B. cereus*, produced limited amount of keratinases and proteases in the presence of pig bristle and wool, while human hair proved to be a favored inducer. Despite notable keratinolytic activity of 12.8 KU, negligible amount of soluble proteins and low concentration of liberated amino acids, up to 0.63 mM, was recorded. Noticeably higher accumulation of hydrolysis products was in cultures of *B. cereus* on each of the substrates, hair, wool and bristle, however, only at wool and bristle significant presence of hydrolytic enzymes was observed. 

## 5. Discussion

Biodegradation of keratins in culture conditions is a process dependent on the microbial strain and the type of the keratin appendage. Relatively short, four-day culture time on hardly degradable substrates is usually sufficient exclusively for bacteria of eminent keratinolytic potential (15, 16). 

Native keratin of epidermis is rarely studied as an inducer in keratinase production. A report from Chao et al. (17) illustrated an optimized method for biosynthesis of a keratinase from *Streptomyces* sp. on native human foot skin medium.

Moreover, higher activity of the produced enzyme against this substrate was highlighted, in comparison to keratin azure, hair and feathers. In the presented study, pig bristle and human hair were subjected to similar extent of biodegradation in short, four-day cultures, however *B. polymyxa* produced more keratinases on hair substrate while *B. cereus* on bristle. It appears that both strains were more adapted to utilization of keratin appendages with thick outer cuticle, which in pig bristle is formed of about 30 cell layers and 10 layers in human hair (18). Despite a single-layer cuticle, lamb wool was not an easily degradable and accessible substrate for the tested bacteria. 

During experiments on *B. licheniformis* K18102, presented by Desai et al. (18), comparative cultures on chicken feathers and other materials like bovine hair, wool, human hair and nails were investigated. The strain produced significant keratinolytic activity in the presence of all substrates, except human hair and nails, measured after thirteen days of culture. The activity on bovine hair and wool was 69.2% and 64.2% of the value obtained in a feather-supplemented medium, respectively. The extent of protein liberation reflected levels of keratinase biosynthesis, reaching 0.85 mg/cm^3^ in the case of feathers and 0.47 mg/cm^3^ in the case of bovine hair. Similar correlation was observed in amino acid concentration in culture media. Human hair and nails proved to be poor keratinase inducers and were more resilient to the biodegradation, as indicated by lower amount of hydrolysis products.

According to Desai et al. (18), wool represented a more digestible substrate for *B. licheniformis* than human hair. In our experiment involving *B. polymyxa *and *B. cereus*, lamb wool was nearly equally resistant to biodegradation and triggered comparable keratinase production, nevertheless it was more beneficial for the *B. cereus* strain. 

Generally, spatial structure of hair-type appendages, especially the presence protective outer cuticle layers, is responsible for their unique resilience, nevertheless natural microbial adaptation to their biodegradation is possible. Prakash et al. (10) investigated *Bacillus* sp. PPKS-2 of unique specificity to human hair as a sole nutrient source. The strain biosynthesized 2.6-9.4% more keratinases on hair or horn meal, respectively, as compared to chicken feathers. Hair was also most beneficial for strains caseinolytic activity. The authors reported that hair was completely decomposed within seven days of culture, while feather material required eight days. Further improvement of keratinolytic activity and biodegradation rate was achieved by application of additional proteinaceous supplements to the culture medium. 

Mazotto et al. (8) presented *B. subtilis* AMR strain also exhibiting immense keratinolytic potential against human hair. After 4 days of culture keratinolytic activity was constantly of growing trend, indicating evident substrate utilization. Moreover, maximum keratinase production was recorded after eight days of culture. Likewise, one of the strains inquired by Lal et al. (19), *B. licheniformis* S23, exhibited great capability of human hair decomposition, especially in prolonged cultures. Maximum keratinase activity was obtained after one month of cultivation and the amount of reduced thiols reached nearly 0.3 mM, proving strains keratinolytic action against hair, in contrast to cow hoove or horn and human nail material. 

As elucidated by Mazotto et al. (8) keratinic substrate pretreatment could be of significant importance in terms of improving keratinase production. Specifically, application of feather meal instead of raw feathers in cultures of two *B. subtilis* strains resulted in evident enhancement of keratinase biosynthesis, accompanied with elevated level of soluble proteins. In contrast, no such relation was observed in the case of another examined strain of *B. licheniformis*. However, only in culture of one strain greater keratinase production was correlated with increased substrate utilization. In terms of effective keratinase production on either of substrates it is crucial to optimize culture conditions. Besides physical parameters, culture medium supplements, mainly protein components and carbohydrate carbon sources, are of highest importance. Among widely used additives yeast extract, peptons, beef extracts and various grain flours or carbohydrates like glucose, sucrose or maltose, can be listed (8, 10, 20).

The results obtained our study clearly confirm keratinolytic nature of both evaluated *Bacillus* strains, which were able to produce keratinases in the presence of diverse keratinic waste. It was illustrated that keratinolytic potential of tested microorganisms, regardless of their significant dissimilarity in degradation mode, was distinctly directed against β-keratin of feathers rather than α-keratin of hair, wool or bristle. Nevertheless, some extent of biodegradation and enzyme production was denoted on either of these keratin types. Despite processes focused on keratinase production or keratin waste digestion require further optimization, strains applicability in prospective feather waste utilization could be concluded.
